# Health Effects of Underground Workspaces cohort: study design and baseline characteristics

**DOI:** 10.4178/epih.e2019025

**Published:** 2019-08-16

**Authors:** Gerard Dunleavy, Thirunavukkarasu Sathish, Nuraini Nazeha, Michael Soljak, Nanthini Visvalingam, Ram Bajpai, Hui Shan Yap, Adam C. Roberts, Thuan Quoc Thach, André Comiran Tonon, Chee Kiong Soh, Georgios Christopoulos, Kei Long Cheung, Hein de Vries, Josip Car

**Affiliations:** 1 Centre for Population Health Sciences, Lee Kong Chian School of Medicine, Nanyang Technological University Singapore, Singapore, Singapore; 2 Population Health Research Institute, McMaster University, Hamilton, ON, Canada; 3 Research institute for Primary Care and Health Sciences, Keele University, Staffordshire, UK; 4 School of Civil and Environmental Engineering, College of Engineering, Nanyang Technological University Singapore, Singapore, Singapore; 5 Laboratório de Cronobiologia e Sono, Porto Alegre Clínicas Hospital (HCPA), Porto Alegre, Brazil; 6 Postgraduate Program in Psychiatry and Behavioral Sciences, Federal University of Rio Grande Do Sul (UFRGS), Porto Alegre, Brazil; 7 Division of Leadership, Management and Organisation, Nanyang Business School, College of Business, Nanyang Technological University Singapore, Singapore, Singapore; 8 Department of Clinical Sciences, College of Health and Life Sciences, Brunel University London, London, UK; 9 Department of Health Promotion, CAPHRI Care and Public Health Research Institute, Maastricht University, Maastricht, The Netherlands

**Keywords:** Workplace, Environmental health, Lifestyle, Cohort studies

## Abstract

The development of underground workspaces is a strategic effort towards healthy urban growth in cities with ever-increasing land scarcity. Despite the growth in underground workspaces, there is limited information regarding the impact of this environment on workers’ health. The Health Effects of Underground Workspaces (HEUW) study is a cohort study that was set up to examine the health effects of working in underground workspaces. In this paper, we describe the rationale for the study, study design, data collection, and baseline characteristics of participants. The HEUW study recruited 464 participants at baseline, of whom 424 (91.4%) were followed-up at 3 months and 334 (72.0%) at 12 months from baseline. We used standardized and validated questionnaires to collect information on socio-demographic and lifestyle characteristics, medical history, family history of chronic diseases, sleep quality, health-related quality of life, chronotype, psychological distress, occupational factors, and comfort levels with indoor environmental quality parameters. Clinical and anthropometric parameters including blood pressure, spirometry, height, weight, and waist and hip circumference were also measured. Biochemical tests of participants’ blood and urine samples were conducted to measure levels of glucose, lipids, and melatonin. We also conducted objective measurements of individuals’ workplace environment, assessing air quality, light intensity, temperature, thermal comfort, and bacterial and fungal counts. The findings this study will help to identify modifiable lifestyle and environmental parameters that are negatively affecting workers’ health. The findings may be used to guide the development of more health-promoting workspaces that attempt to negate any potential deleterious health effects from working in underground workspaces.

## INTRODUCTION

How populations live and work is shifting, with urbanization continuing to increase now that 55% of the world’s population lives in urban areas [1]. Increasingly cities are seeing subterranean development as a strategy to meet the challenge of accommodating a greater population density [2]. Underground spaces can have a wide range of functions, including public use (e.g., shopping centres), personal use (e.g., garages), transportation (e.g., subways), utilities (e.g., water), and storage (e.g., oil), and can also serve as workspaces (e.g., offices) [3]. Although the development of underground workspaces (UWSs) may be seen as part of a solution to healthy urban growth, and as a means to reduce urban sprawl [4], questions remain as to the impact of spending extended periods of time in an UWS on an individual’s health and well-being.

UWSs pose some risks in comparison to aboveground workspaces (AWSs), with a lack of exposure to natural sunlight being the most prominent concern [5,6]. Light is the most significant external factor in synchronizing inner circadian rhythms, which regulate the behaviour, physiology, endocrinology, and metabolism of most living systems [7]. The effect of light on sleep-wake cycles and melatonin secretion is well established [8,9], and several studies have reported that underground environments impact humans’ sleep-wake cycle [10-12]. Circadian rhythm disruption is associated with an increased risk for obesity, diabetes [13], and stroke [14]. Underexposure to natural light has also been reported to negatively impact individuals’ mental health. A number of psychological effects have been reported by those in UWSs, including anxiety [15] and depressive symptoms [16]. These psychological effects may be the result of a lack of natural light and/or a consequence of thoughts about being in an enclosed space; thoughts of confinement were highlighted as a key concern in a survey of over 1,000 participants regarding attitudes towards UWSs [17]. Additionally, indoor air quality may also be an issue in UWSs. High humidity, which is a complaint among workers in UWSs [18], is of concern as it promotes bacterial and fungal growth. A meta-analysis of 33 studies reported an association between the presence of building mould and dampness and the development of upper respiratory tract symptoms, cough, and asthma [19]. Indoor parameters such as humidity and temperature have been shown to be correlated with sick building syndrome [20], and these parameters can be difficult to maintain in UWSs [21]. To date, research on subterranean environments has mostly focused on engineering, and studies of their health effects typically involved limited professions in extreme UWS environments (e.g., miners) [16,22]. Information is limited on the health impacts from working underground in less extreme environments, such as in office-based professions, and how those impacts change over time.

In order to better understand the health effects of UWSs, we established a workplace cohort in Singapore, called the Health Effects of Underground Workspaces (HEUW) cohort, comprising workers from UWSs and AWSs. Our primary objectives are to examine the effects of working in UWSs on sleep quality and melatonin levels. Our secondary objectives are to examine whether the UWS environment has effects on circadian rhythm, vitamin D deficiency, health-related quality of life (HRQoL), psychological distress, sick building syndrome, and lung function.

The aim of this paper is to describe the rationale, study design, data collection, and baseline characteristics of the cohort.

## MATERIALS AND METHODS

### Study design, setting, and recruitment of participants

**Figure 1.**
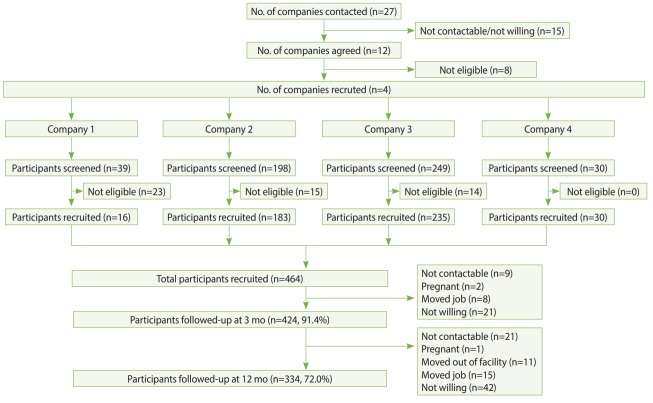
Flowchart showing the selection of study sites and participants, and follow-up.

Recruitment of participants and baseline assessment of the HEUW cohort were conducted from August 2016 to January 2017. UWSs in Singapore were identified through online searches and discussion with civil engineers who were part of the research team. Subsequently, to obtain a suitable comparison group, AWSs with workers with a comparable job type or industry to those in UWSs were identified. A total of 27 companies in Singapore were contacted through personal visits, phone calls, and emails, of which 15 were either uncontactable or unwilling and 8 were small with fewer than 20 employees. In total, 4 companies were recruited including those from the transport industry (n=2), a cooling plant (n=1), and a university (n=1). Recruitment of participants across the 10 sites from these 4 companies was conducted in 2 steps. First, the study team approached the worksites and met with the senior management team to discuss the study. Once confirmation of participation from the management team was obtained, employees were invited to participate via worksite posters, meetings, and emails. Employees expressed their interest through their management team or directly registered with the study team at the recruitment session. Those willing to participate were screened for eligibility. Participants were eligible for selection if they were aged 21 years and above, and worked for at least 4 hr/d at their assigned workspace. Participants were deemed ineligible for selection if they were pregnant or if on average, they made at least 1 trip/mo to countries in a different time zone from Singapore in the past 6 months. Figure 1 shows the selection of study sites and participants and their follow-up at 3 months and 12 months.

### Sample size calculation

We conducted a precision-based sample size calculation for both primary outcomes (sleep quality and melatonin levels). For sleep quality, data (unpublished) from the National Population Health Survey in Singapore [23], an ongoing survey on a representative sample (18-79 years) of Singapore citizens and permanent residents, showed that the average mean Pittsburgh Sleep Quality Index (PSQI) score was 4.12, with a standard deviation (SD) of 2.69. Assuming the true difference in mean PSQI would lie within ±1 unit of the estimated difference with a 95% confidence interval (CI), we needed a minimum of 60 participants from UWSs and 60 from AWSs. At the time of recruitment, there were no published data on melatonin available for the Singaporean population. Therefore, we used data of normative melatonin secretion values from a Japanese study [24], in which the mean± SD was 121.94±123.85 ng/mL. Assuming that the true difference in mean melatonin secretion would lie within ±10 ng/mL and with reported variance of 50% and a 95% CI, we needed a minimum of 128 participants from UWSs and 128 from AWSs. To have a better representation of participants from AWSs, we doubled that sample size to 256. We further adjusted this sample size for a 20% attrition rate at 1-year; hence, the operational sample size for this study was 461 participants. A 1-year follow-up was deemed sufficient, as participants employed in UWSs were already working for a median (interquartile range [IQR]) of 4.2 (2.5 to 8.0) years and those employed in AWSs were working for a median (IQR) of 3.3 (2.2 to 6.5) years at the time of recruitment. Furthermore, a recent systematic review of 15 studies showed that reduced melatonin levels due to exposure to artificial light recovered within 15 minutes after cessation of exposure, indicating that artificial light exposure has short-term effects on melatonin secretion [25].

### Ethics statement

The study was approved by the Institutional Review Board (IRB) of Nanyang Technological University Singapore (IRB-2015-11-028). Study participants provided written informed consent prior to the commencement of data collection.

### Measurements

Table 1 shows the measurement domains, tools, and follow-up time points.

### Questionnaires

Standardized and validated questionnaires were used to collect data on socio-demographic characteristics, health behaviours, work-related characteristics, psychological characteristics, chronotype, HRQoL, medical history, sick building syndrome, and indoor environment quality (IEQ) measures.

#### Socio-demographic characteristics

Data on age, gender (men, women), marital status (never married, divorced, widowed and married), education (primary and secondary, pre-college, and college degree and above), occupation, nationality (Singaporean or foreigner), ethnicity (Chinese, Malay, Indian, or others), housing type (Housing & Development Board flat, condominium, terrace, semi-detached, or bungalow) and monthly income (<S$2,000, S$2,000-S$3,999, S$4,000-S$5,999, S$6,000-S$9,999, ≥S$10,000) were collected.

#### Health behaviours

Data on smoking habits and alcohol drinking were collected using standardized questions from the World Health Organization (WHO) STEPS questionnaire [26]. Smoking questions collected information on lifetime smoking, current smoking, frequency of smoking, and amount of cigarettes smoked. Alcohol questions pertained to frequency of alcohol drinking and the average amount of alcohol consumed on a drinking day. Physical activity (PA) was assessed using the Global Physical Activity Questionnaire [27]. The duration (minutes) of an activity performed during work, travel, and leisure time on a typical day was multiplied by its metabolic-equivalent task (MET) value, and they were summed to obtain the total MET-min/wk. A MET value of 4 was assigned for moderate activities and a MET value of 8 was given for vigorous activities. The total MET-min/wk was used to categorize participants according to their PA levels; low (<600 MET-min/wk), moderate (600-2,999 MET-min/wk), and high (≥3,000 MET-min/wk) [34]. Sedentary behaviour was assessed by the following question: “How much time do you usually spend sitting or reclining on a typical day?” Dietary habits were assessed by a Food Frequency Questionnaire (FFQ), adapted from the FFQ used in the National Population Health Survey in Singapore [23]. The FFQ included questions about the usual intake of a range of food items and drinks over the last 12 months. Data on portion size and frequency of intake of these food items or drinks were collected. Eating behaviour was assessed by asking participants’ dinnertime on weekdays and weekends, and whether they snacked between dinner and bedtime. Sleep quality was measured using the PSQI [28]. This questionnaire has 19 self-rated items grouped into seven components: subjective sleep quality, sleep latency, sleep duration, habitual sleep efficiency, sleep disturbances, use of sleeping medication, and daytime dysfunction. Poor sleep quality was defined as a PSQI score >5 [28].

#### Work-related characteristics

Questions were included in the self-administered questionnaires to ascertain the number of years employed at the current company, work location (UWS or AWS), presence of a window viewable from the participants’ work desk, job type (control room, office, or workshop), daily working hours, shift work (day, afternoon, evening, and night shifts) on a fixed or rotational basis, average number of night shifts in a month, and average hours spent at the work desk in a day.

#### Chronotype

The Morningness-Eveningness Questionnaire (MEQ) was used to assess participants’ chronotype [31]. The questionnaire contains 19 items related to the respondent’s preferred times for waking up and going to bed and daily activity schedules. MEQ scores range from 16 to 86; scores <42 indicate “evening types,” scores >58 indicate “morning types,” and scores between 42 and 58 indicate “intermediate types.”

#### Psychological distress and stress

The General Health Questionnaire-12 (GHQ-12) was used to measure participants psychological distress [30]. The questionnaire contains 12 items, with 4 possible options for each item. The questionnaire includes questions on concentration, sleep, mood, emotions, self-worth, and worries during the previous 4 weeks. Responses range over a 4-point scale, from “less than usual” to “much more than usual”, and the original GHQ scoring method (0-0-1-1) was applied [35]. We applied a cut-off score of >1 to categorize participants with psychological distress [36]. Stress at home, stress at work, and financial stress were each assessed with single-item questions [37]. To assess stress at home and at work, participants were asked: “How often have you felt stress: (1) at work in the past 12 months?; (2) at home in the past 12 months?” Participants could select from: (1) never experience stress; (2) some period of stress; (3) several periods of stress; (4) permanent stress. Financial stress was assessed with the following question: “What level of financial stress do you feel?” Participants could select from: (1) none; (2) little; (3) moderate; (4) high/severe.

#### Health-related quality of life

The Short Form-36v2 (SF-36v2) questionnaire was used to assess HRQoL [29]. The SF-36v2 is a well-validated and widely used generic instrument to measure HRQoL. The SF-36v2 is divided into 8 scales (physical functioning, role limitation-physical, role limitation-emotional, bodily pain, general health, mental health, social functioning, and vitality) and 2 domains (physical component summary and mental component summary). Scores for each scale and domain range from 0 to 100, with higher scores indicating a better quality of life.

#### Medical history

Self-reported comorbidities were assessed using questions on the history of various chronic medical conditions including diabetes, heart disease, stroke, high cholesterol, hypertension, chronic kidney disease, peripheral vascular disease, asthma, allergy, and mental disorders. Participants also reported whether a family member (father, mother, or siblings) had been diagnosed with specific diseases (heart disease, hypertension, diabetes, chronic kidney disease, and dyslipidaemia), and their age of diagnosis of the disease. We also collected information on the regular use of medications and supplements.

#### Sick building syndrome

Sick building syndrome was assessed by a questionnaire that has been used in a nationwide morbidity survey in Singapore [32]. The questionnaire covers 11 symptoms; nose-related (stuffy, runny or sneezing), dry throat, cough, skin rash/itch, eye irritation, headache, fatigue, drowsiness/sleepiness, dizziness, nausea/vomiting, and breathing difficulties. Sick building syndrome was defined as the onset of 2 or more symptoms at least twice weekly while in the building, overnight resolution of these symptoms after leaving the building or workstation, and absence of known medical causes.

#### Indoor environment quality parameters

The OFFICAIR questionnaire was used to assess the perceived comfort levels of indoor environmental conditions (temperature, noise, light, and air) [33]. For each of these parameters, participants were asked: “How would you describe the typical indoor conditions in your office environment during the past month?” These questions were answered on a 7-point scale, ranging from 1 (dissatisfied) to 7 (satisfied).

### Objective measurements

#### Anthropometry

Height, weight, and waist and hip circumference were measured by trained staff in accordance with standard protocols and tools [26]. Height was measured using a stadiometer (Seca 217, Seca GmbH, Hamburg, Germany) to the nearest 0.1 cm, and weight was measured in light clothing using a digital scale (Seca 874, Seca GmbH) to the nearest 0.1 kg. Overweight (body mass index [BMI], 23.0-27.4 kg/m^2^) and obesity (BMI ≥27.5 kg/m^2^) were defined as per the WHO recommendation for Asian populations [38]. Waist and hip circumferences were measured by a stretch-resistant tape (Seca 201, Seca GmbH). Waist circumference was measured at the midpoint between the lower margin of the last palpable rib and the top of the iliac crest (hip bone). Hip circumference was measured at the maximum circumference over the buttocks. Two measures of central obesity were calculated, based on the waist-to-hip ratio (WHR) or waist circumference alone. The WHR was calculated as the ratio between waist and hip circumferences and based on this, we defined central obesity as a WHR of ≥0.90 in men and ≥0.85 in women [39]. Using waist circumference, we defined central obesity as a waist circumference of >0.90 cm in men and >0.80 cm in women [40].

#### Blood pressure

Blood pressure, in accordance with the National Health and Nutrition Examination Survey (NHANES) protocol [41], was measured over the right arm using the appropriate cuff size with an automatic digital blood pressure monitor (Dinamap Pro100V2, Criticon, Norderstedt, Germany). The assessment was conducted by trained staff and 3 readings were taken with 2-minute intervals between the readings.

#### Actigraphy and sleep diary

Participants wore an Actiwatch (Actiwatch Spectrum Plus, Phillips Respironics, Bend, OR, USA), which contains an accelerometer capable of estimating locomotor activity (e.g., movement, rest/activity periods) and a luximeter that assesses ambient light exposure. Participants were requested to wear the Actiwatch 24 hours a day, for 8 consecutive days. Participants were instructed on how to use the device by trained staff and they were also requested to complete a sleep diary. The data were input into the ‘nparACT’ package for R and the chronobiology integrated software ‘El Temps’ (http://www.el-temps.com/principal.html). Double-plotted actograms were created to illustrate rest-activity rhythms. Cosinor analysis was performed by fitting the data to a sinusoidal curve of a 24-hour rhythm, which provided the following variables: mesor, amplitude, and acrophase. A Sokolove and Bushell periodogram was used to analyse the period of activity rhythm for each subject. Non-parametric serial analyses provided intracycle variability (a measure of rhythm fragmentation), interdaily stability (a measure of synchronization of the time series to the 24-hour light/dark cycle), and relative amplitude of data, as well as the 5 hours of lowest levels and the 10 hours of highest values for each variable.

#### Fitness tracker

Participants were requested to wear a Fitbit Charge 2 (Fitbit Inc., San Francisco, CA, USA) 24 hours a day for 23 consecutive days. The device collected information on participants’ steps, distance, calories, heart rate, and sleep.

#### Blood tests

Venous blood samples were collected from participants in a fasting state (at least 8 hours) by trained phlebotomists. A maximum of 11 mL of blood was drawn into 2 tubes – 8 mL in a plain tube and 3 mL in a fluoride tube. Blood samples were transported immediately, in cooler boxes (4°C), to an internationally accredited laboratory for analysis. Samples were processed using the hexokinase method for plasma glucose and enzymatic methods for serum lipids on a Cobas 6000 analyzer, using kits supplied by Roche Diagnostics (Basel, Switzerland). Low-density lipoprotein cholesterol was estimated using the Friedewald equation for those with triglycerides ≤4.52 mmol/L [42], while for the rest, values were estimated by the direct method. Serum 25-hydroxyvitamin D concentrations were measured using the chemiluminescence immunoassay method on a Cobas e 411 analyzer with kits supplied by Roche Diagnostics.

#### Urine tests

We adhered to the NHANES home urine collection manual to collect an overnight urine sample [43]. Ice packs and a Styrofoam box were provided to participants to keep the urine cool overnight. The timing of the first urine void after 8 p.m. and the timing of the first-morning void were recorded. Total urine volume was measured and recorded. Urine samples were sent to the National University Hospital Tissue Repository Laboratory for processing and storage. Urine was aliquotted into twenty 1 mL tubes for storage and processing. One aliquot per sample was sent to National University Hospital Reference Laboratory to run urine cortisol and creatinine tests, and another aliquot from each sample was sent to the Adelaide Research Assay Facility, University of Adelaide for melatonin measurements. Overnight melatonin secretions were estimated by measuring the primary urinary metabolite of melatonin, 6-sulphatoxymelatonin, by double-antibody radioimmunoassay, using standards and reagents supplied by Stockgrand Ltd. (Guildford. UK).

#### Lung function

We followed the NHANES respiratory health spirometry procedures manual to conduct the spirometry tests in this study [44]. Forced expiratory volume in 1 second (FEV1), forced vital capacity (FVC), and the ratio of FEV1 to FVC were determined using an Easy-on PC Spirometer (ndd, Zurich, Switzerland). All spirometry examinations were performed with participants in a sitting position. Each participant was required to perform 3 acceptable manoeuvres. As per the NHANES guidelines, the 2 highest values for FVC and FEV1 needed to demonstrate minimal variability [44].

#### Indoor environment quality measures

IEQ parameters were objectively measured at participants’ work desks or work areas for a period of 10 minutes on a random workday. For instruments (i) to (iii) (see below), individual readings were obtained for participants with individual workspaces (i.e., specific work desks, cubicles, or work stations), whereas 5-10 readings (depending on the size of the workspace) were taken for participants in shared workspaces. The average of those readings was then assigned to participants working in those workspaces. Various instruments were used to measure the different indoor environmental parameters, as follows:

(i) Spectrometer: An optic spectrometer (AvaSpec-ULS2048L StarLine Versatile Fiber-optic Spectrometer) was used to obtain readings of illuminance (lux) at participants’ eye level at their work desks/spaces.
(ii) Digital IEQ meter: A thermal comfort meter (Testo 480, Lenzkirch, Germany), was used to measure air temperature, relative humidity, and air velocity at workplaces.
(iii) Aerosol meter: Particulate air pollution was measured with an aerosol meter (DustTrak DRX Model 8533EP, TSI, Shoreview, MN, USA) for a 10-minute period for each participant.
(iv) Microbial sampling: Microbial air sampling was carried out according to the SS554: 2016 guideline for Good Indoor Air Quality in Office Premises [45]. Single-stage microbial viable impactor sampling using the Surface Air System principle was used as a tool to collect and concentrate air in order to identify the microbial quality of the air. Triplicate readings of each selected sampling point of a workspace were measured for a period of 10 minutes at 3 different time points in a day. Laboratory analysis of air samples was conducted by a laboratory accredited under the Singapore Laboratory Accreditation Scheme.

#### Psychological and social measures

A number of psychological parameters were assessed, including personality characteristics, decision-making, sustained attention, response inhibition, global or local precedence, perseverance, abstract reasoning, working memory, attention, and effort discounting (Supplementary Material 1). Unlike the health measures, the majority of the psychological tests were conducted as one-off measures. Computer tests were conducted using Mueller & Piper’s Psychological Experiment Building Language [46].

### Baseline characteristics

Tables 2-6 show the baseline characteristics of participants of the cohort (n=464).

Socio-demographic characteristics: The mean age of participants was 39.0±11.4 years, with a large proportion (40.9%) aged more than 40 years. The majority were men (79.5%), were of Chinese ethnicity (63.8%), were married (60.3%), had at least post-secondary education (89.5%), and earned <S$4,000 per month (71.3%). There was a higher percentage of men working in UWSs than in AWSs; this was the only significant demographic difference between groups (Table 2).

Health behaviours, stress, psychological distress, HROoL, and chronotype: Nearly a quarter of participants were current smokers (24.4%) and engaged in low levels of PA (23.1%), and slightly more than half (53.4%) were alcohol drinkers. Two-thirds (66.0%) consumed fruits and vegetables below the WHO-recommended levels (i.e., <5 servings/d). A large proportion had poor sleep quality (42.5%), close to two-thirds (62.3%) had experienced stress at home in the past 12 months, three-quarters (75.4%) were currently having financial stress, and 24.4% were considered to be experiencing psychological distress. The mean HRQoL scores for the physical and mental health scales were 51.6 and 50.2, respectively. In terms of chronotype, almost one-quarter of participants were morning types (22.4%), whilst the majority were intermediate types (65.9%) and the remaining were evening types (11.6%). There were no significant differences in health behaviours, stress, psychological distress, HROoL, or chronotype between those working in either workspace (Table 3).

Anthropometric and clinical measurements: Based on BMI, more than two-thirds (67.0%) of participants were either overweight or obese. Almost 39.2% and 34.5% of participants had central obesity based on waist circumference and the WHR, respectively. There were no significant differences in anthropometric or clinical measurements between those working in either workspace (Table 4).

Work-related characteristics: Nearly one-third (30.6%) were working in UWSs, and the median duration of employment was 3.8 years. The majority were office workers (48.5%), followed by control room staff (30.2%) and workshop staff (21.3%). The mean working duration per day was 8.6 hours, while more than one-third (35.8%) were shift workers. More than four-fifths (82.8%) had experienced work stress in the past 12 months. Almost one-fifth (17.9%) reported experiencing sick building syndrome symptoms because of their workspace. The only work-related characteristic that differed between groups was working hours, with individuals working in UWSs working an average of 36 minutes longer per day (Table 5).

IEQ measures: The overall satisfaction levels with light, temperature, noise, and air quality were high, with scores ranging from 4.5 for air quality to 4.9 for light. Those working underground were significantly less satisfied with the artificial lighting in their workspace. Lux levels were below the recommended level of 500 lux in AWSs and UWSs [47]; however, there was no difference in lux between AWSs and UWSs (Table 6).

## KEY FINDINGS

The first paper from this cohort study was recently published [48], and found that there was no difference in the prevalence of sick building syndrome between participants in under and AWSs. Additional studies from this cohort study are undergoing peer review at various journals, and will subsequently be published.

## STRENGTHS AND WEAKNESSES

Strengths of our study include a reasonably large sample size for a workplace cohort, the use of standardized and validated questionnaires, and objective measurements of a wide range of clinical, biochemical and environmental parameters. A unique strength of this research is the multi-disciplinary approach undertaken, comprising health, psychological, and social measures. We also had high levels of questionnaire data completeness with less than 1% of missing data for variables.

Our study is not without limitations. There was an over-representation of men, as the industries comprised mainly positions generally taken up by men such as engineers, technicians, and traffic controllers. Attrition is a common issue in workplace studies. There was a 28% loss to follow-up at 1-year, mainly due to staff turnover and a lack of time owing to work commitments or work shifts. Comparable rates of attrition have been observed in other longitudinal workplace studies in Asia at follow-up periods similar to our study [49,50]. We could not measure biochemical parameters at baseline due to logistical issues with regard to vendors and equipment. Objective environmental measurements were made difficult by work disruption and nature of work; thus, only 10 minutes of recording was possible on random workdays, which may not have accurately reflected the workplace’s environmental parameters.

## DATA ACCESSIBILITY

The study data are not freely available, but the study team would welcome collaborations with other researchers and data sharing is possible upon request and ethics approval. For further information, contact Associate Professor Josip Car, Director of the Centre for Population Health Sciences, Lee Kong Chian School of Medicine, Nanyang Technological University Singapore (josip.car@ntu.edu.sg).

## Figures and Tables

**Table 1.** Health measurements, tools, and data collection time-points

Component	Measurement tools/questions	Baseline	3 mo	12 mo
Socio-demographic, lifestyle, medical history, health and work-related measurements and tools				
Socio-demographic characteristics	Age, gender, ethnicity, occupation, nationality, marital status, monthly income, and housing	√	√	√
Alcohol consumption and smoking	WHO STEPS questionnaire [26]	√	√	√
Diet	FFQ adapted from the FFQ used in the National Population Health Survey, Singa- pore [23]	√	√	√
Physical activity and sedentary behaviour	Global Physical Activity Questionnaire [27]	√	√	√
	Steps, distance, calories, heart rate, and sleep duration with Fitbit Charge 2 (Fitbit Inc., San Francisco, CA, USA)	×	×	√
Sleep quality	Pittsburgh Sleep Quality Index [28]	√	√	√
Comorbidities	History of high cholesterol, diabetes, stroke, coronary heart disease, mental health disorders, hypertension, peripheral vascular disease, asthma, and allergy	√	√	√
Medication use	Regular use of medications and supplements	√	√	√
Family history	Family history of high cholesterol, diabetes, coronary heart disease, chronic kidney disease, and hypertension	√	√	√
Work-related characteristics	Work location (aboveground or underground workspace), presence of a window, no. of work hr/d, shift work, duration of employment in the current company, and job type (office, control room, or workshop)	√	√	√
Health-related quality of life	36-item Short Form Health Survey [29]	√	√	√
Stress	Likert scale (4-point) on experiences of stress at work, at home, and financial stress [35]	√	√	√
Psychological distress	General Health Questionnaire-12 [30]	√	√	√
Circadian rhythm (light exposure and locomotor activity)	Mesor, amplitude, acrophase, intracycle variability, interdaily stability, and relative amplitude (Actiwatch Spectrum Plus, Phillips Respironics, Bend, OR, USA)	×	×	√
Chronotype	Morningness–Eveningness Questionnaire [31]	√	√	√
Sick building syndrome	11-item questionnaire [32]	√	√	√
Anthropometric and clinical measurements and tools				
Weight	Seca digital scale (Seca 874, Seca GmbH, Hamburg, Germany)	√	√	√
Height	Seca stadiometer (Seca 217, Seca GmbH, Hamburg, Germany)	√	×	×
Waist and hip circumference	Seca measuring tape (Seca 201, Seca GmbH, Hamburg, Germany)	√	√	√
BP	Digital BP monitor (Dinamap Pro100V2 Criticon, Norderstedt, Germany)	×	×	√
Blood tests (pathology)	Fasting plasma glucose, lipids, and 25-hydroxyvitamin D	×	×	√
Urine tests (pathology)	Melatonin (6-sulphatoxymelatonin)	×	×	√
Spirometry	Forced expiratory volume in 1 second and forced vital capacity with Easy-on PC Spirometer (ndd, Zurich, Switzerland)	√	×	√
Indoor environmental quality measurements and tools				
Light exposure	Lux (AvaSpec-ULS2048L StarLine Versatile Fiber-optic Spectrometer, The Netherlands)	×	×	√
	Dominant wavelength (AvaSpec-ULS2048L StarLine Versatile Fiber-optic Spectrometer, The Netherlands)			
	Lux (Actiwatch Spectrum Plus, Phillips Respironics, Bend, OR, USA)	×	×	√
Self-perceived environmental quality	European project OFFICAIR questionnaire covering thermal comfort, variation in temperature, air movement, noise, light, and vibration [33]	√	√	√
PM	PM_1_, PM_2.5_, PM_4_, and PM_10_ (DustTrak DRX Model 8533EP, TSI, Shoreview, MN, USA)	×	×	√
Thermal comfort	Predicted percentage dissatisfied, predicted mean vote, temperature, humidity, and carbon dioxide with a thermal comfort meter (Testo 480, Lenzkirch, Germany)	×	×	√
Bacterial and fungal counts	Single-stage microbial viable impactor sampling using Surface Air System	×	×	√

WHO, World Health Organization; FFQ, Food Frequency Questionnaire; BP, blood pressure; PM, particulate matter.

**Table 2.** Socio-demographic characteristics of the study cohort at baseline

Characteristics	Total (n=464)	Aboveground (n=322)	Underground (n=142)	p-value^1^
Age (yr)				
Mean±SD	39.0±11.4	38.8±11.4	39.6±11.4	0.494
21-30	153 (33.0)	109 (33.8)	44 (31.0)	0.800
31-40	121 (26.1)	84 (26.1)	37 (26.0)	
>40	190 (40.9)	129 (40.1)	61 (43.0)	
Gender				0.044
Men	369 (79.5)	248 (77.0)	121 (85.2)	
Women	95 (20.5)	74 (23.0)	21 (14.8)	
Ethnicity				0.493
Chinese	296 (63.8)	204 (63.4)	92 (64.8)	
Malays	99 (21.3)	73 (22.7)	26 (18.3)	
Indians	48 (10.3)	33 (10.2)	15 (10.6)	
Others^2^	21 (4.5)	12 (3.7)	9 (6.3)	
Marital status				0.495
Single^3^	184 (39.7)	131 (40.7)	53 (37.3)	
Married	280 (60.3)	191 (59.3)	89 (62.7)	
Education				0.536
Primary and secondary	49 (10.6)	33 (10.2)	16 (11.3)	
Pre-college	250 (53.9)	179 (55.6)	71 (50.0)	
College and above	165 (35.6)	110 (34.2)	55 (38.7)	
Monthly income (S$)				0.773
<4,000	331 (71.3)	231 (71.7)	100 (70.4)	
≥4,000	133 (28.7)	91 (28.3)	42 (29.6)	

Values are presented as number (%). SD, standard deviation.

1 Student t-test for normally distributed continuous variables and the Pearson chi-square test for categorical variables.

2 Includes mixed ethnicities, Indonesians, Pakistanis, and Filipinos.

3 Includes never-married, widowed, divorced, and separated.

**Table 3.** Health behaviours, stress, psychological distress, health-related quality of life, and chronotype of the study cohort at baseline

Characteristics	Total (n=464)	Aboveground (n=322)	Underground (n=142)	p-value^1^
Smoking status				0.829
Never smoked	303 (65.3)	208 (64.6)	95 (66.9)	
Ex-smoker	48 (10.3)	35 (10.9)	13 (9.2)	
Current smoker	113 (24.4)	79 (24.5)	34 (23.9)	
No. of cigarettes smoked/d (among current smokers)	6.0 [1.4-10.0]	4.3 [0.5-10.0]	7.1 [1.4-10.0]	0.192
Alcohol drinking				0.382
Non-drinker	216 (46.6)	153 (47.5)	63 (44.4)	
Drinks less than once a month	161 (34.7)	114 (35.4)	47 (33.1)	
Drinks once or more than once a month	87 (18.7)	55 (17.1)	32 (22.5)	
No. of standard drinks of alcohol/drinking day (among alcohol drinkers)	2 [1-3]	2 [1-3]	2 [1-3]	0.910
Physical activity				0.525
Low	107 (23.1)	79 (24.5)	28 (19.7)	
Moderate	200 (43.1)	136 (29.3)	64 (45.1)	
High	157 (33.8)	107 (33.2)	50 (35.2)	
Sedentary time (hr/d)	6.7±3.7	6.6±3.7	6.9±3.6	0.466
Fruit and vegetables servings/d	3.6 [2.2-5.6]	3.6 [2.2-5.8]	3.6 [2.2-5.6]	0.506
PSQI global score	5.5±2.8	5.4±2.8	5.6±2.7	0.574
Poor sleep quality (PSQI score >5)	197 (42.5)	136 (42.2)	61 (43.0)	0.787
Stress at home in the previous 12 mo				0.272
Never experienced stress	175 (37.7)	129 (40.1)	46 (32.4)	
Some periods of stress	253 (54.5)	168 (52.2)	85 (59.9)	
Several periods of stress/permanent stress	36 (7.8)	25 (7.8)	11 (7.7)	
Current level of financial stress				0.486
None	114 (24.6)	76 (23.6)	38 (26.8)	
Little	222 (47.8)	160 (49.7)	62 (43.7)	
Moderate or severe	128 (27.6)	86 (26.7)	42 (29.6)	
GHQ-12 score	0 [0-1]	0 [0-1]	0 [0-2]	0.434
Psychological distress (GHQ-12 score >1)	113 (24.4)	76 (23.6)	37 (26.1)	0.570
Physical component summary score of HRQoL scale	51.6±6.7	51.6±6.7	51.6±6.7	0.977
Mental component summary score of HRQoL scale	50.2±7.7	50.5±7.7	49.5±7.8	0.225
Chronotype				0.492
Morning	104 (22.4)	77 (23.9)	27 (19.0)	
Intermediate	306 (65.9)	209 (64.9)	97 (68.3)	
Evening	54 (11.6)	36 (11.2)	18 (12.7)	

Values are presented as mean ± standard deviation (normally distributed) or median [interquartile range] (skewed) for continuous variables, and number (%) for categorical variables. PSQI, Pittsburgh Sleep Quality Index; GHQ, General Health Questionnaire; HRQoL, health-related quality of life.

1 Student t-test for normally distributed continuous variables, the Wilcoxon rank-sum test for non-normally distributed continuous variables, and the Pearson chi-square test for categorical variables.

**Table 4.** Anthropometric and clinical measurements of the study cohort at baseline

Characteristics	Total (n=464)	Aboveground (n=322)	Underground (n=142)	p-value^1^
Weight (kg)	72.8±17.2	73±17.5	72.5±16.3	0.771
Body mass index (kg/m^2^)	25.6±5.2	25.8±5.4	23.3±4.9	0.414
Body mass index categories (kg/m^2^)				0.666
Underweight or normal (<23.0)	153 (33.0)	101 (31.4)	52 (36.6)	
Overweight (23.0-27.4)	191 (41.1)	138 (42.9)	53 (37.3)	
Obesity (≥27.5)	120 (25.9)	83 (25.8)	37 (26.1)	
Waist circumference (cm)	85.9±13.3	85.8±13.5	86.2±13.1	0.737
Hip circumference (cm)	99.1±9.6	99.0±10.1	99.2±8.7	0.839
Waist-to-hip ratio	0.86±0.07	0.86±0.07	0.87±0.07	0.723
Central obesity (based on waist circumference)	182 (39.2)	125 (38.8)	57 (40.1)	0.788
Central obesity (based on waist-to-hip ratio)	160 (34.5)	113 (35.1)	47 (33.1)	0.677

Values are presented as mean±standard deviation (normally distributed) or number (%) for categorical variables.

1 Student t-test for normally distributed continuous variables and the Pearson chi-square test for categorical variables.

**Table 5.** Work-related characteristics of the study cohort at baseline

Characteristics	Total (n=464)	Aboveground (n=322)	Underground (n=142)	p-value^1^
Years based at work location	3.8 [2.3-6.8]	3.3 [2.2-6.5]	4.2 [2.5-8.0]	0.068
Job type				0.881
Control room worker	140 (30.2)	99 (30.7)	41 (28.9)	
Office worker	225 (48.5)	156 (48.4)	69 (48.6)	
Workshop worker	99 (21.3)	67 (20.8)	32 (22.5)	
Work (hr/d)	8.6±1.3	8.4±1.0	9.0±1.7	<0.001
Shift work				0.193
No	298 (64.2)	213 (66.1)	85 (59.9)	
Yes	166 (35.8)	109 (33.8)	57 (40.1)	
Night shift				0.748
No	325 (70.0)	227 (70.5)	98 (69.0)	
Yes	139 (30.0)	95 (29.5)	44 (31.0)	
Average night shifts/month (among night shift workers)	8.2±3.7	7.8±3.7	9.1±3.7	0.050
Work stress in the previous 12 mo				0.500
Never experienced stress	80 (17.2)	57 (17.7)	23 (16.2)	
Some periods of stress	279 (60.1)	197 (61.2)	82 (57.8)	
Several periods of stress or permanent stress	105 (22.6)	68 (21.1)	37 (26.0)	
Sick building syndrome	83 (17.9)	60 (18.6)	23 (16.2)	0.528

Values are presented as mean±standard deviation (normally distributed) or median [interquartile range] (skewed) for continuous variables, and number (%) for categorical variables.

1 Student t-test for normally distributed continuous variables, the Wilcoxon rank-sum test for non-normally distributed continuous variables, and the Pearson chi-square test for categorical variables.

**Table 6.** Indoor environmental parameters of the study cohort at baseline

Characteristics	Total (n=464)	Aboveground (n=322)	Underground (n=142)	p-value^1^
Objective environmental measures				
Illuminance (lux)^2^	123.7±75.4	126.5±82.2	116.9±54.6	0.233
Subjective indoor environment measures				
Overall comfort	4.9±1.2	4.9±1.1	4.8±1.2	0.559
Light overall	4.9±1.2	4.9±1.2	4.8±1.2	0.239
Thermal comfort	4.7±1.3	4.6±1.4	4.8±1.3	0.094
Noise overall	4.8±1.4	4.8±1.3	4.9±1.4	0.352
Air quality overall	4.5±1.3	4.5±1.3	4.4±1.3	0.751
Detailed subjective indoor environment measures				
Light				
Artificial light	5.0±1.2	3.7±1.9	3.1±1.8	0.002
Natural light	3.5±1.9	5.0±1.2	5.0±1.2	0.903
Reflection or glare to no reflection or glare	4.8±1.3	4.7±1.3	4.9±1.3	0.136
Temperature				
Temperature varies	5.7±1.7	5.8±1.7	5.6±1.8	0.159
Too cold or too hot	5.4±1.7	5.4±1.7	5.4±1.6	0.813
Air quality				
Smelly or odourless air	4.6±1.3	4.6±1.2	4.6±1.3	0.966
Humid or dry air	5.5±1.6	5.6±1.6	5.4±1.7	0.259
Stuffy or fresh air	3.9±1.2	4.0±1.2	3.7±1.2	0.051
Air movement	5.2±1.8	5.3±1.7	5.0±1.9	0.071
Noise and vibration				
Noise from outside the building	5.1±1.5	5.0±1.4	5.2±1.5	0.241
Noise from building systems	4.9±1.4	4.8±1.3	4.9±1.4	0.794
Noise from sources other than building systems	4.7±1.4	4.6±1.4	4.8±1.4	0.117
Vibration	5.1±1.4	5.1±1.4	5.1±1.4	0.709

Values are presented as mean±standard deviation.

1 Student t-test for normally distributed continuous variables and the Wilcoxon rank-sum test for non-normally distributed continuous variables.

2 Measurements taken for 430 participants.
