# Supplementary material for: Health Effects of Underground Workspaces cohort: study design and baseline characteristics
Source: Epidemiol Health. 2019 Aug 16;41:e2019025. doi: 10.4178/epih.e2019025 (PMC6815877; doi:10.4178/epih.e2019025)
Supplement: Supplementary file 1 [file epih-41-e2019025-suppl.docx]

Supplementary material 1: Psychological and social measures

| *Component* | *Measure (Assessment point)* | *Description* |
| --- | --- | --- |
| *Decision Making* | Prisoner’s Dilemma (PD) Game(1)  (Assessment 1) | Prisoner’s Dilemma (PD) Game examines cooperative vs competitive/defective behaviours when an individual has the option to make a decision that will affect the other party (in our study, it was another human participant).  The PD used in our study was framed as a situation of monetary gain for both parties. |
|  | Temporal Discounting Task(2) (Assessment 2) | The task was framed as investing in a business opportunity which provide a smaller return the very next day or a larger return but after one/three/six months.  Hyperbolic discounting model values were used(2). |
|  | Public Goods (PG) Game(3)  (Assessment 2) | Public Goods (PG) game was modified from Gunnthorsdottir et al. (2007). PG examines cooperative (interest of the group) vs competitive (self-interest) behaviours.  Participants were given $10 cash and were informed that they can take any amount for themselves and/or return any amount to participant which will be added into public goods. Additional money will be based on the amount of public goods received and then shared amongst all members of the group – i.e. their colleagues participating). |
| *Personality Characteristics* | Behavioural Activation/Inhibition System (BAS/BIS) Scale(4) (Assessment 1) | According to Reinforcement Sensitivity Theory (RST) proposed by Gray, BIS is sensitive to punishment and inhibits behaviour that might result in negative outcomes. Hence, BIS activation results in inhibition of movement towards goals. BAS, however, is sensitive to rewards and escape from punishment. Thus, activation of BAS results in movement or an increase of movement towards goals.  Measures for BAS system were divided into three subscales: Pleasure Seeking (tendency to seek out new rewarding experiences), Drive (strong pursuit of goals) and Reward Responsiveness. |
|  | 10-item Big Five Inventory(5)  (Assessment 1) | Big Five Inventory has been widely used in research as a measure for personality. The five personality traits refers to Openness (i.e. openness to experience), Extraversion, Agreeableness, Conscientiousness and Neuroticism.  For our study, a shorter version of the Big Five Inventory was used. It had 10 items, measured on a 5-point Likert scale. |
|  | Locus of Control (Internality) Scale(6)  (Assessment 1)  Locus of Control (Chance) Scale(6)  (Assessment 2) | Locus of control (LOC) examines how an individual attribute causes or reasons for their experiences (success and/or failures).  The original LOC scale, examines three different aspects of LOC: internality, chance and powerful others. For the study, we examined only two aspects; internality (individual’s belief that events/things are within their control) and chance (individual’s belief that events/things happen by luck or chance). Each aspect had 8 items that used 5-point Likert scale. Some questions were slightly modified so they fit the general population better (original scale was created based on medical theme). |
|  | Highly Sensitive Person (HSP) scale(7)  (Assessment 2) | This scale measures an individual’s sensory processing sensitivity. An individual with high-sensitive personality will be more aware and sensitive to the changes in their environment. Hence, these individuals are more likely to be overwhelmed by sensations in their environment.  For the study, we used a 12-item scale, measured on a 7-point Likert scale(8). |
| *Cultural Orientation/*  *Values* | Horizontal and vertical individualism and collectivism scale(9)  (Assessment 1) | Most cultural studies looks into individualism-collectivism orientation; where individualist tends to value interest of self above the group’s, whereas collectivist is the opposite, prioritizing group’s interest over self.  Horizontal-vertical orientation looks at the preference for existence of ranks (vertical) or parity (horizontal) within the culture.  This scale looks at these four cultural orientations: Horizontal Individualism, Horizontal Collectivism, Vertical Individualism, Vertical Collectivism. This scale contains 14 items that used 7-point Likert scale. |
|  | Uncertainty Avoidance Scale (10)  (Assessment 1) | CVSCALE is a scale that looks into cultural values of an individual. For our study, only a sub-section of CVSCALE, uncertainty avoidance, was included.  Uncertainty avoidance is the degree where individual feels that they are vulnerable by ambiguous situations. The uncertainty avoidance sub-scale had 5 items measured on 5-point Likert scale. |
| *Perception regarding environment* | (Assessment 1) | In order to gain a better understand of our participant’s current working environment, our team added several questions that required participant to rate the importance and satisfaction of various elements (like lighting, background noise, etc.) of their current working environment.  Additionally, there were several questions that aimed to understand how important nature was for the individual. |
| *Claustrophobia* | Claustrophobia Scale(11)  (Assessment 1) | This scale examines the fear of being in enclosed spaces.  For the study, participants were given 4 situations and were asked to rate how anxious they felt in each situation on a 5-point scale ranging from not at all anxious to very anxious. |
| *Organisational Measures – Job Satisfaction and Turnover Intent* | Michigan Organisational Assessment Questionnaire - Turnover Intent subscale(12)  (Assessment 1, 2 and 3) | Turnover intent looks into an employee’s intention to voluntarily leave their current position at their organisation.  This scale, created by Cammann, Fichman, Henkins and Klesh (1979) (13) was taken from Wong, Wong and Wong, 2015(12), had a total of 3 questions, measured on a 5-point Likert scale. |
|  | Michigan Organisational Assessment Questionnaire - Job Satisfaction subscale(13)  (Assessment 1 and 2) | Job satisfaction looks into an employee’s satisfaction with their current job.  This scale had a total of 3 questions, measured on a 7-point Likert scale. |
| *Work Fatigue* | 3-Dimensional Work Fatigue Scale (14)  (Assessment 1, 2 and 3) | Frone and Tidwell (2015) defined work fatigue as “extreme tiredness and reduced functional capacity that is experienced during and at the end of the workday”.  This scale looks into three different types of work fatigue (physical, mental and emotional) at the end of each workday. For each dimension, there were 6 items asking participant to rate the frequency that they experience fatigue after each workday in the past month, on scale ranging from never experience to experience after every workday. |
| *Attitudes towards working in underground spaces* | Underground workspaces questionnaire(15) (Assessment 2) | This questionnaire allows us to examine what kind of attitudes, expectations and views an individual can have for underground workspaces.  This scale explores 3 types of views that one may have for underground spaces: perception of calmness and safety, negative affect and confinement.  In total, the scale contained 16 items that were measured on a 7-point Likert scale. |
| *Abstract Reasoning* | Raven’s Standard Progressive Matrices(RSPM)(16)  (Assessment 2) | RSPM is a multiple-choice test often used to assess mental ability associated with abstract reasoning (fluid intelligence). It has little dependency on language abilities as it is a pattern matching task. |
| *Alexithymia* | Toronto Alexithymia Scale(17)  (Assessment 3) | Alexithymia refers to individuals that have issues identifying and/or describing how they feel and find it hard to focus their attention externally.  The 20 items questionnaire was measured on a 5-point Likert scale ranging. |
| *Autism spectrum conditions* | Autism Spectrum Quotient(18)  (Assessment 3) | Characteristics of autism spectrum conditions includes having issues interacting or communicating with others, adjusting to changes, displaying the same behaviours repeatedly and more.  For the study, a questionnaire was included examine if there were individuals with such conditions in our sample.  This questionnaire had a total of 10 items, measured using a 4-point scale ranging from strongly agree to strongly disagree. The items were scored using binary scoring. |
| *Work Characteristics* | Work Design Questionnaire(19) (Assessment 3) | For the study, our participants were adults from the working population. Hence, a work characteristics questionnaire was used to gain a better understanding of their work.  The questionnaire examines four main types of characteristics (task, knowledge, social and contextual) of a job.  The questionnaire had a total of 77 items, measured using 5-point Likert scale. |
| *Sustained Attention* | Psychomotor Vigilance Task (PVT)(20) (Assessment 1) | PVT is a simple sustained attention reaction time task.  Participants were instructed to respond as soon as they can when a visual stimulus appeared on the screen. Their responses were tabulated into 4 different groups – false alarm, correct, lapse and sleep. The task was set to last for 3 minutes in total. Each participant, on average, completed around 20-25 trials (depending on how fast they were). |
| *Response Inhibition* | Error Monitoring Psychological Response Task(21) (Assessment 1) | This task measures response inhibition and sustained attention. There were two stimulus, one with high frequency (letter P) while the other had low frequency (letter R).  Participants were instructed to respond if a particular stimulus (like letter P) appear while inhibiting their response for the other stimulus (letter R) [P-Go, R-NoGo trials]. Subsequently, the letters were switched over [R-Go, P-NoGo trials]. There were two blocks of trials. Each block had 10 practice trials and 160 actual testing trials. |
| *Global or Local Precedence* | Navon Local-Global Task (22) (Assessment 1) | This task uses an adaptation of Navon figures (1977) to examine global or local precedence when processing visual stimuli.  For the task, there were seven different types of trials (total of 150 trials) that participants had to complete. It began with single stimulus (local/global type). Subsequently, the trials contained combination of both types of features (local stimulus with global distractor or global stimulus with local distractor).  There were two different letters involved in the task – H and S. |
| *Attention and Perseverance* | Change Blindness (CB) Task  (Assessment 2) | CB measures attention, visual search ability and the willingness to persevere to complete something that seemed difficult or impossible to complete. The task used was a modified version of Rensink, O’Regan and Clark’s experiment (1997)(23).  Participants were instructed to identify a change between two flickering images. The change might occur in the foreground or background of the images. There were some images that did not have any difference between them.  There were 15 pictures in total for the task. |
| *Working Memory* | Digit Span Task(24)  (Assessment 3) | Digit Span Task measures the working memory capacity of individuals. For the task, participants were supposed to remember the order of digit presentation and type out the digits presented after the entire presentation was completed.  The “staircase” type digit span task was used for our study. In the event that the participant answered correctly, the next trial will have one more digit presented. Otherwise, the next trial will have one less digit. There were a total of sixteen trials. |
| *Attention and Effort discounting* | Letter Counting Task (LCT) and Effort Discounting (ED) Task(25)  (Assessment 3) | LCT measures attention placed on small details. For this task, participants were given a paragraph of nonsensical words and were instructed to count the number of times a particular letter appeared.  ED looks at the amount of effort required to complete a particular task by using monetary incentive as a measure. Individual’s value of the monetary incentive is likely to decline if the effort required to obtain the incentive is increased.  Participants were given different monetary incentives and asked how much incentive had to be offered before they were willing to repeat LCT once/twice/thrice. Both tasks were conducted simultaneously. |

#### References

1. Capraro V, Jordan JJ, Rand DG. Heuristics guide the implementation of social preferences in one-shot Prisoner's Dilemma experiments. Scientific Reports. 2014;4:6790. doi: 10.1038/srep06790.

2. Mazur JE. An adjusting procedure for studying delayed reinforcement. The effect of delay and of intervening events on reinforcement value. Quantitative analyses of behavior, Vol. 5. Hillsdale, NJ, US: Lawrence Erlbaum Associates, Inc; 1987. p. 55-73.

3. Gunnthorsdottir A, Houser, D., & McCabe, K. Disposition, history and contributions in public goods experiments. Journal of Economic Behavior & Organization. 2007;62(2):304-15. doi: 10.1016/j.jebo.2005.03.008.

4. Carver CS, & White, T. L. Behavioral inhibition, behavioral activation, and affective responses to impending reward and punishment: the BIS/BAS scales. Journal of personality and social psychology. 1994;67(2):319.

5. Rammstedt B, John OP. Measuring personality in one minute or less: A 10-item short version of the Big Five Inventory in English and German. Journal of Research in Personality. 2007;41(1):203-12. doi: 10.1016/j.jrp.2006.02.001.

6. Levenson H. Multidimensional locus of control in psychiatric patients. Journal of consulting and clinical psychology. 1973;41(3):397-404. Epub 1973/12/01. PubMed PMID: 4803272.

7. Aron EN, Aron A. Sensory-processing sensitivity and its relation to introversion and emotionality. J Pers Soc Psychol. 1997;73(2):345-68. Epub 1997/08/01. PubMed PMID: 9248053.

8. M. P. Sensory-processing sensitivity: a potential mechanism of differential susceptibility. 2013.

9. Sivadas E, Bruvold NT, Nelson MR. A reduced version of the horizontal and vertical individualism and collectivism scale: A four-country assessment. Journal of Business Research. 2008;61(3):201-10. doi: 10.1016/j.jbusres.2007.06.016.

10. Yoo B, Donthu N, Lenartowicz T. Measuring Hofstede's Five Dimensions of Cultural Values at the Individual Level: Development and Validation of CVSCALE. Journal of International Consumer Marketing. 2011;23(3-4):193-210. doi: 10.1080/08961530.2011.578059.

11. Ost LG. The claustrophobia scale: a psychometric evaluation. Behaviour research and therapy. 2007;45(5):1053-64. Epub 2007/02/17. doi: 10.1016/j.brat.2004.10.004. PubMed PMID: 17303070.

12. Wong YT, Wong, Y. W., & Wong, C. S. An integrative model of turnover intention: Antecedents and their effects on employee performance in Chinese joint ventures. Journal of Chinese Human Resource Management. 2015;6(1):71-90.

13. Cammann C, Fichman, M., Jenkins, D., & Klesh, J. The Michigan Organizational Assessment Questionnaire. University of Michigan, Ann Arbor. . [Unpublished manuscript]. In press 1979.

14. Frone MR, Tidwell MO. The meaning and measurement of work fatigue: Development and evaluation of the Three-Dimensional Work Fatigue Inventory (3D-WFI). Journal of occupational health psychology. 2015;20(3):273-88. Epub 2015/01/21. doi: 10.1037/a0038700.

15. Lee EH, Luo C, Sam YL, Roberts AC, Kwok KW, Car J, et al. The underground workspaces questionnaire (UWSQ): Investigating public attitudes toward working in underground spaces. Building and Environment. 2019;153:28-34. doi: <https://doi.org/10.1016/j.buildenv.2019.02.017>.

16. Bilker WB, Hansen JA, Brensinger CM, Richard J, Gur RE, Gur RC. Development of abbreviated nine-item forms of the Raven's standard progressive matrices test. Assessment. 2012;19(3):354-69. Epub 2012/05/19. doi: 10.1177/1073191112446655.

17. Bagby RM, Parker JD, Taylor GJ. The twenty-item Toronto Alexithymia Scale--I. Item selection and cross-validation of the factor structure. Journal of psychosomatic research. 1994;38(1):23-32. Epub 1994/01/01. PubMed PMID: 8126686.

18. Allison C, Auyeung B, Baron-Cohen S. Toward brief "Red Flags" for autism screening: The Short Autism Spectrum Quotient and the Short Quantitative Checklist for Autism in toddlers in 1,000 cases and 3,000 controls [corrected]. Journal of the American Academy of Child and Adolescent Psychiatry. 2012;51(2):202-12.e7. Epub 2012/01/24. doi: 10.1016/j.jaac.2011.11.003. PubMed PMID: 22265366.

19. Morgeson FP, Humphrey SE. The Work Design Questionnaire (WDQ): developing and validating a comprehensive measure for assessing job design and the nature of work. The Journal of applied psychology. 2006;91(6):1321-39. Epub 2006/11/15. doi: 10.1037/0021-9010.91.6.1321. PubMed PMID: 17100487.

20. Dinges DF, Powell JW. Microcomputer analyses of performance on a portable, simple visual RT task during sustained operations. Behavior Research Methods, Instruments, & Computers. 1985;17(6):652-5. doi: 10.3758/BF03200977.

21. Bezdjian S, Baker LA, Lozano DI, Raine A. Assessing inattention and impulsivity in children during the Go/NoGo task. The British journal of developmental psychology. 2009;27(Pt 2):365-83. doi: 10.1348/026151008X314919. PubMed PMID: 19812711.

22. Navon D. Forest before trees: The precedence of global features in visual perception. Cognitive Psychology. 1977;9(3):353-83. doi: <https://doi.org/10.1016/0010-0285(77)90012-3>.

23. Rensink RA, O'Regan JK, Clark JJ. To see or not to see: The need for attention to perceive changes in scenes. Psychological Science. 1997;8(5):368-73. doi: 10.1111/j.1467-9280.1997.tb00427.x.

24. Croschere J, Dupey, L., Hilliard, M., Koehn, H., & Mayra, K. . The effects of time of day and practice on cognitive abilities: Forward and backward Corsi block test and digit span. In PEBL Technical Report Series [Online], #2012-03: 2012.

25. Kurzban R, Duckworth A, Kable JW, Myers J. An opportunity cost model of subjective effort and task performance. The Behavioral and brain sciences. 2013;36(6):661-79. doi: 10.1017/S0140525X12003196. PubMed PMID: 24304775.
